# Linear and non-linear responses of vegetation and soils to glacial-interglacial climate change in a Mediterranean refuge

**DOI:** 10.1038/s41598-017-08101-y

**Published:** 2017-08-14

**Authors:** Jens Holtvoeth, Hendrik Vogel, Verushka Valsecchi, Katja Lindhorst, Stefan Schouten, Bernd Wagner, George A. Wolff

**Affiliations:** 1 University of Liverpool, School of Environmental Sciences, Liverpool, UK; 2 University of Bristol, School of Chemistry, Bristol, UK; 3 University of Cologne, Institute of Geology and Mineralogy, Cologne, Germany; 4 University of Bern, Institute of Geological Sciences & Oeschger Centre for Climate Change Research, Bern, Switzerland; 5 13 Rue du Cannau, Montpellier, France; 6 University of Kiel, Institute of Geosciences, Kiel, Germany; 7 NIOZ Royal Netherlands Institute for Sea Research, Department of Marine Microbiology and Biogeochemistry, Den Burg, Texel The Netherlands; 8 University of Utrecht, Department of Earth Sciences, Utrecht, The Netherlands

## Abstract

The impact of past global climate change on local terrestrial ecosystems and their vegetation and soil organic matter (OM) pools is often non-linear and poorly constrained. To address this, we investigated the response of a temperate habitat influenced by global climate change in a key glacial refuge, Lake Ohrid (Albania, Macedonia). We applied independent geochemical and palynological proxies to a sedimentary archive from the lake over the penultimate glacial-interglacial transition (MIS 6–5) and the following interglacial (MIS 5e-c), targeting lake surface temperature as an indicator of regional climatic development and the supply of pollen and biomarkers from the vegetation and soil OM pools to determine local habitat response. Climate fluctuations strongly influenced the ecosystem, however, lake level controls the extent of terrace surfaces between the shoreline and mountain slopes and hence local vegetation, soil development and OM export to the lake sediments. There were two phases of transgressional soil erosion from terrace surfaces during lake-level rise in the MIS 6–5 transition that led to habitat loss for the locally dominant pine vegetation as the terraces drowned. Our observations confirm that catchment morphology plays a key role in providing refuges with low groundwater depth and stable soils during variable climate.

## Introduction

Lake sediment records are important environmental archives that record the response of terrestrial habitats to global climate change^1, 2^. Broad-scale changes in vegetation and lake productivity in response to glacial-interglacial transitions have been studied in a range of lacustrine settings^3–7^. However, lakes may not necessarily respond in a coherent manner within a region; global and regional trends can be overwhelmed by local aspects of environmental response such as changes in lake level, nutrient fluxes or soil stability^7^. In this context, the dynamics of the quantitatively important soil carbon pool within lacustrine catchments often remain elusive, due to the heterogeneous nature of the soil cover and its dependence on local geology and morphology^8–10^. Novel approaches using ^14^C compound-specific isotope analysis show promise in distinguishing delivery of contemporary vs. older OM stored in soils and later eroded material^11^, but these are not appropriate for records older than *ca*. 50 ka.

**Figure 1:**
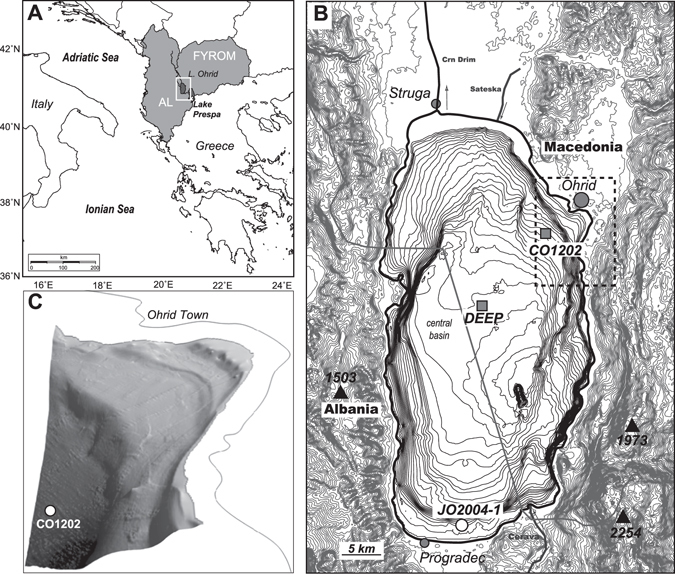
Location of Lake Ohrid in the Western Balkans (**A**), morphology and bathymetry of the Ohrid Basin and position of study site Co1202, the ICDP coring site DEEP and core JO2004-1﻿ (**B**) and high-resolution bathymetry of the terrace system in Ohrid Bay (**C**). Plates A-C were generated using Generic Mapping Tools (GMT 5.2.1; http://gmt.soest.hawaii.edu), Global Mapper (http://www.bluemarblegeo.com) and Fledermaus 7.0 (http://www.qps.nl), respectively.

Here, we use a combined organic geochemical and palynological approach to investigate a sediment record from Lake Ohrid (site Co1202, Fig. 1C), a trans-boundary lake shared between Albania and Macedonia (Fig. 1A,B; area 358 km^2^, maximum depth 293 m, average depth 155 m)^12–15^ that, together with other intramontane basins in the Western Balkans, formed a key glacial refuge area for deciduous trees^16–18^. During the penultimate glacial-interglacial transition (Termination II), there was broad-scale vegetation change in this region^19, 20^, which is at the boundary between Mediterranean and north European climatic influence. Trees commonly dominated during warm and moist interglacial conditions, while herbs and grasses were dominant during the cold and dry glacial climate^20^.

The sediment record covers the period from 140 to ﻿99 ka B.P., in particular, the transition from the penultimate glacial, *i*.*e*. marine isotope stage (MIS) 6 to the last peak interglacial, MIS 5e, and the following interstadial (5d) and stadial (5c), including the North Atlantic cold events C24 and C23 (112–108.8 and 105.1–102.6 ka, respectively)^21^. The lake is surrounded by mountain ranges (1500–2200 m.a.s.l.) and, with no large river entering the basin, its direct catchment is small (1310 km^2^)^22^. Hence, processes occurring in the immediate surroundings of the basin control the inputs of terrestrial OM to the lake sediments as well as nutrient supply for aquatic biomass production.

Lake level fluctuated substantially over the course of MIS 6 and 5, with evidence for phases of regression and transgression exposing or flooding subaquatic terraces at 12, 30 and 60 m below present-day water depth (Fig. 1C)^14^. The lowest lake level of about 60 m below present occurred in MIS 6, but earlier than the oldest sediment recovered at Co1202. It remains uncertain if the lake-level highstands during MIS 5 exceeded modern lake level. Sedimentological^11^ and archaeological findings^23^ such as the remains of Neolithic pile dwellings behind the modern shoreline in the modern town of Ohrid certainly suggest that lake level was higher than present earlier in the Holocene, shaping the floodplains above modern lake level (Fig. 1B,C) and this may well have been the case during MIS 5. While Co1202 remained at substantial water depth (currently 145 m) over the course of MIS 6 and 5, parts of the shoreline would have moved closer to the study site, exposing relatively flat surfaces between the lake and the mountain slopes. We demonstrate how it is possible to disentangle the interplay between global climate change, vegetation and soil stock dynamics and how these were influenced by local factors, particularly lake level and morphology.

## Choice of Proxies

Lake surface temperature (LST) changes are primarily controlled by changes in air temperature^24^. We use the TEX_86_ molecular proxy^25, 26^ (see Supplementary Materials for details) and the amount of endogenic calcite (CaCO_3_)^13^ deposited in the sediments to determine relative changes in LST at Lake Ohrid. Changes in the vegetation structure are reconstructed through palynological analysis focussing on arboreal (trees plus shrubs) pollen types, in particular *Pinus* spp. (pine), *Quercus* spp. (oak, deciduous and evergreen) and *Abies* spp. (fir). For the Ohrid Basin, it has been shown that the ACL FA_22–26_ (average chain length of fatty acids between C_22_ and C_26_) is a proxy for relative input of soil OM *vs*. leaf litter, and that the FA/OH_25–30_ (the ratio of fatty acids to alcohols with carbon numbers 25 to 30) reflects the degradation state of soil OM^27^ (see Supplementary Material for further explanation of the proxies). Distributions of alkyl lipids together with bulk^28^ and compound-specific isotope data (see Fig. S2 and Supplementary Materials) suggest that soil OM dominates the lake sediments, with little contribution from phytoplankton or macrophytes.

## Results and Discussion

### Local climate development

**Figure 2:**
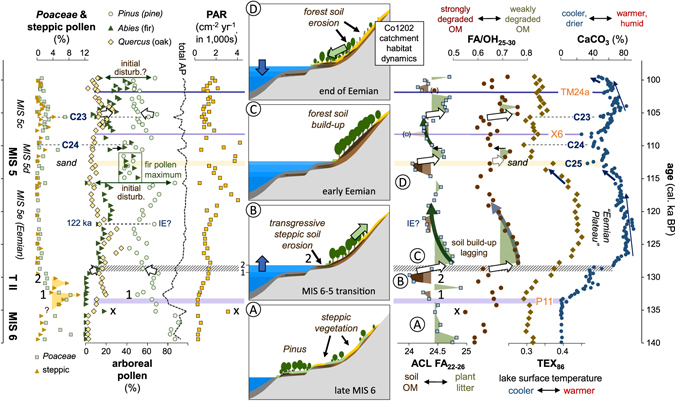
Records of palynological and organic geochemical data from site Co1202 (Ohrid Bay) from 140 to 99 thousand calendar years before present (cal. ka BP; BP = 1950) and schematic model of lake-level and habitat dynamics across the aridity-humidity cycle from ~137 to ~112 ka (insets **A**–**D**). *Quercus* spp. includes deciduous and evergreen species, with the latter typically accounting for less than 5% of the total *Quercus* pollen. Slightly elevated evergreen proportions (>10%, max. 19%) occur between 115 and 111 ka; PAR = pollen accumulation rate (grains cm^−2^ yr^−1^). CaCO_3_ data has been published previously^13^. Blue horizontal lines labelled P11, X6 and TM24a represent tephra layers used to establish the age model of Co1202^13, 38^; MIS = marine isotope stage, T II = Termination II (MIS 6–5 transition), C23–25 = North Atlantic cold events 23–25 corresponding to the Central European Montaigu, Melisey 1 and Woillard events (see text for references), IE = Intra-Eemian cold event; white arrows mark abrupt shifts in OM quality that represent the reduction of the soil OM pool relative to standing vegetation through terrace drowning or exposure and related vegetation changes; numbers 1 and 2 label the two phases of transgression across the terrace systems in Ohrid Bay and associated soil erosion (brown minima in ACL FA_22–26_), reducing the extent of pine habitat (phase 1) and delivering steppic pollen from the preceding vegetation cover (phase 2, maximum in steppic pollen; inset **B**).

The LST proxies CaCO_3_ and TEX_86_ (Fig. 2) indicate a cold MIS 6 in the Ohrid Basin, with warming commencing at ~133 ka. Subsequently, there was a relatively stable and warm climate during the full interglacial conditions of MIS 5e, with a temperature maximum around 124 ka. A return to colder conditions occurred during MIS 5d, with cooling starting between 116 and 115 ka, followed by a somewhat muted warm phase during MIS 5c.

Short and lower amplitude cold episodes around 110 and 105 ka most likely correspond to the North Atlantic cold events C24 and C23, respectively^29^. The latter coincides with aeolian silt deposition in Central Europe, known as the Montaigu Event, while C24 overlaps with the Melisey 1 stadial^19, 30, 31^. Minima in the CaCO_3_ record also occur at 112.7, 108.3 and 102.0 ka and largely result from the deposition of dated tephras X6 (108.3 ka) and TM24a (102.0 ka) and a supply of sand (112.7 ka) diluting the CaCO_3_. The deposition of the sandy layer probably reflects a shift of the shoreline during a lake-level fall, arising from the Atlantic cold event C25^19, 31^. This can be regarded as the regional expression and culmination of the pronounced cooling trend and associated aridification starting at about 115 ka. Overall, the climatic development in the Ohrid Basin appears in agreement with the general pattern of climatic development recorded in the Northeast Atlantic domain and in central Europe^29, 32, 33^ (see also Supplementary Material).

### Pollen supply

Trends in the pollen profile of Co1202 are generally consistent with pollen records from the south^34^ and centre^35^ of Lake Ohrid, showing an increase in arboreal pollen (Fig. S1 in Supplementary Materials; total aboreal pollen excludes *Pinus*, *Betula and Juniperus* species) during the warm moist Eemian, relative to the cool, dry MIS-6 as seen in numerous other palynological records documenting the adjustment of the continental biomes over Central and Southern Europe^29, 36, 37^. However, between 140 and 135 ka, (MIS 6), the relative amount of pine pollen is higher (80–90%) at Co1202 than reported from the basin centre (40–80%)^35^ or the southern margin (pine + juniper: 20–70%)^34^. At the latter, pollen from steppic species and grasses was substantially more abundant (20–40% vs. <10% at Co1202). Thus, the local vegetation cover across the basin seems to have differed; in the southern basin, close to low-lying hills to the southeast (Fig. 1B) and south, there may have been areas suitable for steppic vegetation, while the catchment of Co1202 in the northeast featured steep mountain slopes as well as low-lying plains (terrace surfaces/flood plains, Fig. 1B,C) that apparently supported a larger stock of pine.

At Co1202, the amount of deciduous and evergreen oak pollen started to rise from about 135 ka (Fig. 2), with the evergreen species averaging at 5% of the total oak pollen. The onset of this vegetation change was marked by a short-term increase in fir pollen of almost 20%, which caused an equivalent drop in the relative amount of pine pollen, while grass and steppic pollen were absent (sample “x” in Fig. 2). This “event” is characterised by the supply of highly degraded OM (very low FA/OH_25–30_; Fig. 2; see following chapter) and a distinct peak in the pollen accumulation rate (PAR) and likely results from the re-activation of parts of the drainage system due to higher rainfall, including influx of soil-stored fir pollen. This first significant change in pollen composition led the temperature change as indicated by the LST proxy records (CaCO_3_, TEX_86_) by almost 2,000 years, but is consistent with recent sedimentological evidence for changes in hydrology and associated lake-level rise occurring throughout the later stages of MIS 6^38^.

At 133 and 131 ka, two distinct peaks in steppic pollen supply (Fig. 2) appear out of phase with the general climatic and basin-wide vegetation development, as they occur during the transition towards warmer and more humid climatic conditions. The first peak appears driven mainly by the reduction in pine pollen supply, as is the concomitant shift towards higher amounts of oak pollen, and coincides with the onset of temperature increase and supply of soil OM (phase 1 in Fig. 2). The second peak occurs mid-way through the MIS 6–5 transition, where there is clear evidence for substantial soil erosion as indicated by biomarker proxies and the input of siliciclastic material diluting the carbonate (see following chapter). The steppic pollen is therefore likely to be part of the soil fraction re-suspended from an upper terrace surface during lacustrine transgression, while oak and pine pollen derived from the contemporary vegetation cover. The high-amplitude shift in the biomarker and CaCO_3_ records at 129 ka that indicates a sharp reduction in the supply of degraded soil-derived material coincided with a further drop in pine pollen supply, implying that another source area for the latter had disappeared. It seems, therefore, that *Pinus* spp. habitats were successively removed from low-lying terrace surfaces close to Co1202 through the rise in lake level during the late MIS 6 and into MIS 5^38^. This led to relatively increased contributions first from steppic vegetation and oaks as the lowermost terrace drowned, with steppic vegetation remaining on the upper terraces and slopes (Fig. 2; phase 1), and then from expanding arboreal vegetation, including pine, and steppic pollen stored in eroding soils as a higher terrace drowned (Fig. 2; phase 2). Similar sharp changes in pollen composition are associated with phases of lower-amplitude climatic change in the younger record, for example, at the termination of the C23 cold event. This may also be explained by lake-level controlled modifications of the terrestrial habitat that altered the proportion of terrace and floodplain surface area relative to the mountain slopes and, hence, the distribution and relative proportions of associated vegetation. Lake-level change does not appear a factor in the modification of the terrestrial habitat in the Co1202 catchment during the mid and late Eemian, i.e. from 123 to 117 ka, where a gradual decline in oak and increase in pine pollen is apparent, or after ~103 ka (MIS 5c) during the gradual expansion of fir. In both cases, the gradual trends in the pollen records reflect the response of the vegetation towards gradual cooling as indicated by the LST proxies.

The onset of accelerated cooling at the end of the Eemian (116 ka) is marked by abrupt changes in pine and fir pollen supply (Fig. 2) initially favouring pine, followed by a 30% increase in fir pollen supply which maximised at this time. Whether or not temporarily enhanced PAR at 118–117 ka, which coincides with a drop in fir pollen and enhanced pine pollen supply, are related to an earlier deterioration of the montane habitat is unclear. The onset of a similar change to the one observed at 116 ka, with accelerating cooling and a pulse in pine peak pollen, appears at the end of the record at 99 ka.

### Organic matter pools and interpretation of the sediment record

The locally calibrated proxies ACL FA_22–26_ and FA/OH_25–30_ (Fig. 2) reflect changes in the quality of terrestrial OM supplied to site Co1202, with lower values indicating enhanced contributions from soil relative to plant litter and more degraded relative to less degraded OM, respectively^27^. They show a seesaw-type pattern and bear little resemblance to the LST records, but rather imply abrupt changes in lipid supply from strongly to less degraded soil OM and plant litter followed by gradual increases in the contribution of degraded OM (Fig. 2). This pattern suggests a threshold-controlled depositional system that nevertheless responds to continuous climate development, since the abrupt changes in OM supply are, in nearly all cases, associated with either major (MIS 6–5 transition) or minor (C23–25) climate events. Considering the terraced morphology within the catchment of Co1202, lake-level rise would have provided a mechanism for such a pattern of change in OM sources, equivalent to some of the abrupt changes in pollen supply. For example, one of the most pronounced changes in OM quality at phase 2 in Fig. 2, as observed in the ACL FA_22–26_ and FA/OH_25–30_ profiles, is associated with the beginning and end of the episode of decreased CaCO_3_ deposition, reflecting dilution with siliciclastic material derived from soil erosion (the concentration of biogenic silica over the whole record never exceeds 8%^13^). The end of phase 2 (~129 ka) is characterised by a switch towards the supply of more litter-type OM, with low amounts of root-derived material (higher ACL FA_22–26_) and less degraded fatty acids (high FA/OH_25–30_).

Given these observations of changing OM quality as indicators of soil erosion, it is possible to synthesise the palynological and geochemical proxy records across the MIS 6–5 transition and to interpret the sediment record. From 140–135.2 ky B.P., pine dominated the catchment of Co1202, perhaps with grassy undergrowth or open grasslands, while steppic vegetation remained in the background (Fig. 2, inset A). At 135.2 ka, oak habitats began to expand, accompanied by an initial activation of the drainage system as indicated by the pulse of highly degraded soil OM (minimum in FA/OH_25–30_) and a stand-alone peak in fir pollen (Fig. 2). This is consistent with the continual hydrological change during this period^38^ and precedes the rapid rise in temperature starting at 133 ka, at the earliest (increase in CaCO_3_). At 133 ka, the pine-dominated habitat on the lowest exposed terrace was drowned (drop in pine pollen), accompanied by transgressive erosion of soils (Fig. 2: phase “1”, minimum in ACL FA_22–26_) and resulting in a relative increase in oak, grass and steppic pollen supply. The supply of soil OM and steppic pollen then reduced for about 1,000 years around 129 ka due to a slow-down in transgressive soil erosion (increased CaCO_3_, ACL_22–26_ FA, FA/OH_25–30_) while lake-level rose across the slope of the next higher terrace and pine successively replaced steppic vegetation (increase in pine pollen, substantial decrease in steppic pollen, small relative decrease in oak pollen). Between 131 and 129 ka, transgressive erosion of more degraded soils from the upper terrace is evident (phase “2”, minima in biomarker proxies and CaCO_3_; see inset B in Fig. 2) that apparently also supplied significant amounts of soil-stored pollen from the former steppic vegetation. By 127.8 ka, the lake level had maximised with all terraces drowned, so plant litter from the slopes became the main source of OM (inset C in Fig. 2). At this stage, a substantial soil pool on the slopes had not yet developed.

Over the following 10,000 years, the supply of degraded soil OM (FA/OH_25–30_) and subsurface biomass (ACL FA_22–26_) steadily increased as soils accumulated under forest vegetation. This is illustrated by the decreasing trends in FA/OH_25–30_ and ACL FA_22–26_ (Fig. 2). There was a gradual decline in oak and increase in pine pollen supply between 128 and 116 ka suggesting vegetation change in response to steady Eemian cooling, as observed in several southern and central European Eemain pollen records^39^, which was potentially disrupted by the Intra-Eemian 122 ka cold climate anomaly^40^. With more severe climate deterioration setting in at 116 ka BP, a significant vegetation change is first indicated by a pulse in pine pollen supply followed by a shift towards substantially higher fir and an equivalent drop in pine pollen supply. From 114.9 ka, soil erosion appeared to increase and substantial amounts of soil OM reached site Co1202 by 113 ka (minimum in ACL FA_22–26_). This is consistent with a significant drop in lake level (inset D in Fig. 2) bringing the shoreline closer to the site^14^ as indicated by the supply of sandy material and eroded former lake sediments from the terrace surfaces that apparently also contributed GDGTs carrying an elevated LST signal from the preceding lake-level highstand (increased TEX_86_). This episode probably relates to the C25 cold event and marks the end of MIS 5e, or the “Eemian”, in our record.

Following this highly dynamic change, the ecosystem briefly stabilised, with reduced soil erosion (switch to higher ACL FA_22–26_ and FA/OH_25–30_) and vegetation establishing on the lowest terrace exposed at the time. There were only minor fluctuations in pollen supply between 115 ka and the onset of the C24 cold event after 111 ka even though soil OM supply had changed dramatically during C25, suggesting that soil OM and pollen derived from the same habitats and that the relative proportions of these did not change significantly. The proportions did change over the course of the C24 event and coincided with the supply of slightly more degraded OM, suggesting that a pine-dominated habitat is destabilised. Between C24 and C23, the carbonate record suggests warming towards MIS 5c, while the pollen records and FA/OH_25–30_ show a similar pattern as in phase 2 in the MIS 6–5 transition, with a small peak in steppic pollen supply coinciding with a drop in pine pollen supply and an increase in OM degradation. Assuming that lake level was rising, following the lowstand at 113 ka, these fluctuations probably resulted from lake-level controlled habitat modifications. At the end of C23, another rapid change in OM quality and LST is recorded, in a similar way to the development of the MIS 6–5 transition, albeit with lower amplitude and without disruption, which suggests that from 113 to 105 ka lake-level rise may have drowned only one terrace level.

## Conclusions

Records of carbonate precipitation and biomarker-based lake surface water temperature (TEX_86_) reflect the climatic development during the last glacial-interglacial transition and marine isotope stages (MIS) 5c-e (140–99 ka B.P.) at Lake Ohrid and reveal a strong relation to NE Atlantic climate (see Supplementary Material for NE Atlantic isotope record). By contrast, due to the location of sediment core Co1202 and its vicinity to a sequence of extended terrace systems, the records of biomarker composition and pollen supply are to a significant extent shaped by local terrestrial habitat dynamics in the catchment, specifically, the exposure and drowning of terrace surfaces. The distributions of different types of soil cover appear crucial in the supply of OM as reflected in the biomarker records. It appears that the terrace surfaces of the Ohrid Basin allowed forest vegetation to persist through periods of falling lake level in areas with low groundwater depth and stable soil cover, in contrast to the basin as a whole where steppic vegetation expanded to much larger extent during arid climatic conditions. Our approach demonstrates that combining proxies sensitive to vegetation as well as soil pool changes enables detailed reconstruction of terrestrial habitat dynamics and underlines the importance of soils as OM sources to environmental archives.

## Material and Methods

### The sedimentary archive of site Co1202

The sediment sequence of site Co1202 is a 15 m composite record of piston cores taken in 2007 from 145 m water depth in the north-eastern part of Lake Ohrid (41° 5′36.91″N; 20°46′2.93″E) about 2.5 km from the shoreline. The sediments are formed of clayey to sandy silts. The stratigraphy of Co1202 is based on tephrochronology and radiocarbon dates that have been described in detail elsewhere^13^. The error for the ages of the TM24a and X6 tephra layers is ±2 ka. The age of the P11 tephra, the oldest in the Co1202 sediment sequence, has recently been reassessed as 133.5 ± 2 ka B.P. as well as the identity of the TM24a tephra^38^. The age model used in this study has been adjusted accordingly.

### Elemental analysis

The determination of CaCO_3_ and total organic carbon (TOC, see Supplementary Materials) have been described and the data reported previously^13^.

### Biomarkers and pollen

Lipid biomarkers were extracted from freeze-dried and homogenised sediment samples by accelerated solvent extraction (ASE) and analysed by liquid chromatography-mass spectrometry (LC-MS, for GDGTs) and gas chromatography-mass spectrometry (GC-MS, for alkyl lipids). For pollen analyses, samples (1 cm^3^) with added tablets of *Lycopodium* spores were treated with HCl (30% v/v) for the removal of carbonates, KOH (10% w/v) for the removal of organic debris, HF (40% v/v) for the removal of silicates followed by acetolysis. Detailed methodologies are given in the Supplementary Material.

### Data availability

The proxy data generated during this study can be found in appendices to the Supplementary Materials. All primary data generated are available through the PANGAEA data repository, https://www.pangaea.de.

## Electronic supplementary material

Supplementary Materials
