# Supplementary material for: Linear and non-linear responses of vegetation and soils to glacial-interglacial climate change in a Mediterranean refuge
Source: Sci Rep. 2017 Aug 14;7:8121. doi: 10.1038/s41598-017-08101-y (PMC5556017; doi:10.1038/s41598-017-08101-y)
Supplement: Supplementary file 1 — Supplementary Materials [file 41598_2017_8101_MOESM1_ESM.pdf]

Supplementary Materials

Holtvoeth, J.*, H. Vogel, V. Valsecchi, K. Lindhorst, S. Schouten, B. Wagner, and G. A. Wolff, 2017.

Linear and non-linear responses of vegetation and soils to glacial-interglacial climate change in a Mediterranean refuge.

*corresponding author

A. Materials and methods

The sedimentary archive of site Co1202

The sediment sequence of site Co1202 is a 15 m composite record of piston cores taken in 2007 from 145 m water depth in the north-eastern part of Lake Ohrid (41° 5'36.91"N; 20°46'2.93"E) about 2.5 km from the shoreline. The sediments are formed of clayey to sandy silts. The stratigraphy of Co1202 is based on tephrochronology and radiocarbon dates and has been described in detail elsewhere[1]. The age of the P11 tephra, the oldest in the Co1202 sediment sequence, has recently been reassessed as 133.5 ± 2 ka B.P. as has the identity of the TM24a tephra, formerly determined as X5[2]. The age model used in this study has been adjusted accordingly.

Elemental analysis

Freeze-dried and homogenized samples were analysed for their elemental composition at centennial resolution of about 200 years, on average. Concentrations of total carbon (TC) were determined by a Vario Micro Cube combustion CNS elemental analyser (VARIO Co.). Total organic carbon (TOC) contents were measured after acid digestion of carbonate (10 % HCl at 80°C) and analysed using a Leco CS-225 carbon–sulfur detector (LECO Corp.). The amount of calcite ($CaCO_3$) was calculated from the difference between TC and TOC, following the equation

$$[CaCO_3] = ([TC] – TOC]) * M_{CaCO3}/M_C \text{ (M = molar mass; } CaCO_3 = 100, C = 12) \quad (1)$$

and assuming that all inorganic carbon is bound in $CaCO_3$.

Lipid extraction and analysis (LC-MS, GC-MS)

5α(H)-cholestane was added as internal standard to splits of the freeze-dried and homogenized sediment samples (5 g) which were then extracted using an organic solvent mixture of dichloromethane (DCM) and methanol (9:1, v:v; 10 mL) and accelerated solvent extraction (ASE; Dionex^{TM}). The bulk of the solvent was removed by rotary evaporation at 30°C.

a) LC-MS analysis of glycerol dibiphytanyl glycerol tetraethers (GDGTs)

For GDGT analyses, aliquots of the total lipid extracts (TLEs) were separated into aliphatic, aromatic and hetero-compound fractions by column chromatography using 5 % deactivated SiO_2 (mesh size 60) and elution with *n*-hexane, followed by DCM/*n*-hexane (2:1, v:v) and methanol, respectively. The solvents were subsequently removed under N_2 gas flow. The methanol (polar) fraction, containing GDGTs, was re-dissolved in *n*-hexane/isopropanol (99:1, v:v) and filtered using a 0.4 µm, 4 mm diameter PTFE filter prior to analysis with a HP 1100 series liquid chromatography–mass spectrometry system (LC-MS) equipped with an auto-

injector and ChemStation chromatography software[3]. Separation was achieved on an Alltech Prevail Cyano column (2.1 x 150 mm; 3 µm) maintained at 30 °C. For the first 5 min, elution was isocratic with 90% A (*n*-hexane) and 10% B (*n*-hexane/isopropanol 9:1, v:v), followed by a linear gradient to 16% B over 34 min. The injection volume of the sample was 10 µL. The mass spectrometer was operated in single ion monitoring mode ($[M + H]^+$).

The TetraEther indeX of tetraethers consisting of 86 carbon atoms (TEX_{86}) and the Branched *vs.* Isoprenoid Tetraether index (BIT) are thought to be proxies for ambient lake surface temperature (TEX_{86}) and input of soil OM (BIT), respectively, and were calculated following the initial publications[4,5]. TEX_{86} is based on the observation that the number of cyclopentane moieties in specific isoprenoidal archaeal membrane lipids (glycerol dibiphytanyl glycerol tetraethers, GDGTs) correlates to temperature during biosynthesis and can thus be used to determine fluctuations in lake surface water temperature. Changes in growth temperature are expressed in the ratio of GDGTs with 1 to 4 cyclopentane moieties, originally introduced as the TEX_{86} [4]. As the communities of GDGT-producing archaea and the input of soil derived GDGT's vary significantly between ecosystems, translating the TEX_{86} ratio into absolute temperature values requires a surface water/surface sediment calibration and evaluation of soil derived GDGT input over time. A TEX_{86} temperature calibration has not yet been carried out for Lake Ohrid and we therefore report the TEX_{86} ratio as such. The BIT is based on the observation that branched, as opposed to isoprenoidal, GDGTs are predominant components of soil organic matter[5,6]. Hence, low (high) BIT values indicate low (high) contributions of soil OM. However, soil OM also contains small amounts of isoprenoidal GDGT's used for the calculation of the TEX_{86}. Therefore, simultaneous analysis of the TEX_{86} and the BIT is commonly applied in order to better understand the bias of soil/terrestrial derived GDGT's on the TEX_{86} signature[7]; bias of the TEX_{86} by terrestrially derived GDGT's is negligible when the BIT is < 0.3. BIT values range between 0.13 – 0.27 (Fig. S1) in the investigated sediment sequence, so we assume that the TEX_{86} values primarily reflect changes in LST.

b) GC-MS analysis of alkyl lipids

Fatty acids were transmethylated by adding acetyl chloride in methanol (1:30, v:v; 1 mL; 0°C) to the TLEs and warming the samples to 45°C for 12 hours. The TLEs were then dried under nitrogen, re-dissolved in DCM and passed through a Pasteur pipette packed with potassium carbonate to remove excess reagent. In order to derivatise compounds with hydroxy groups such as the *n*-alcohols, N,O-bis-(trimethylsilyl)-trifluoroacetamide with 1% trimethylchlorosilane was added to the TLE which was kept at 65°C for 30 minutes prior to analysis.

Aliquots of TLEs in DCM were injected into a Trace 2000 Series gas chromatograph (GC) fitted with an on-column injector and a fused high-temperature silica column (60 m × 0.25 mm i.d.; film: (5 % phenyl-) methylpolysiloxane; DB5-HT equivalent; J&W) with helium as the carrier gas (ca. 1.6 mL min^{-1}). The oven was programmed from 60°C to 170°C at 6°C min^{-1} after 1 minute, then to 315°C at 2.5°C min^{-1} and held for 10 minutes. The GC column was fed directly into the EI source of a Thermoquest Finnigan TSQ7000 mass spectrometer (ionisation potential 70 eV; source temperature 315 °C; trap current 300 µA), operated in Full Data Acquisition mode. Target compounds were identified by their mass spectra and by comparison with authentic standards. Quantitative data were calculated by comparison of peak areas from total ion counts (TIC) of the internal standard with those of the compounds of interest. The relative response factors of the analytes were individually calculated for 36

representative compounds (*n*-fatty acids, unsaturated fatty acids, *n*-alcohols, sterols) based on the analysis of authentic standards. For analytes for which authentic standards were not available, the response factors for similar compounds of the same class and/or similar structure were used. Reproducibility of similar lipid analyses was determined to be < ±15 %[8]. Data quality was checked regularly with procedural blanks for each batch of samples and organic contamination was subtracted from the sample values, although it typically was insignificant (<< 1 % of the sample values).

Compound-specific carbon isotope analysis

Stable carbon isotopic compositions of individual lipids were determined in duplicate or triplicate using a Thermo Trace Ultra gas chromatograph linked by a ConFlo IV interface to a Delta V Advantage isotope ratio monitoring mass spectrometer (irmMS; Thermo Fisher Scientific). Samples were injected in splitless mode onto a DB-5 fused silica capillary column (30 m, 0.25 mm ID, 0.25 µm film thickness, J&W Scientific). The injector temperature was 280ºC. The GC temperature was held at 45ºC for 1 minute, then programmed to 295ºC at 6ºC min^{-1} and held for 15 minutes. The carrier gas was ultra-high purity grade helium (flow: 1.4 mL min^{-1}). The software (Thermo Isodat 3) automatically computed the $^{13}C/^{12}C$ ratios of each compound peak, referenced to a standard gas (CO_2) of known composition. The results are reported in per mil (‰) relative to the VPDB international standard. Standards containing fatty acid methyl esters (Schimmelmann, Indiana University, IN, USA) were used to determine instrument precision (< 0.3 ‰) and accuracy (< 0.5 ‰).

Pollen analysis

A total of 64 sediment samples were chosen for pollen analysis from the studied core sections. *Lycopodium* tablets were added for estimates of pollen concentration[9]. The samples were treated with standard palynological methods[10], including the removal of carbonates, organic debris and silicates and acetolysis. Pollen, spores and palynomorphs were counted at a magnification of × 400 and × 1000 and identified with keys and pollen atlases[11–13]. On average, 520 terrestrial pollen grains were counted. Steppic pollen includes *Chenopodiaceae*, *Artemisia*, *Ephedra distachya* type and *Ephedra fragilis* type. Total arboreal pollen (AP) includes the genera: *Abies*, *Acer*, *Alnus*, *Betula*, *Buxus*, *Carpinus*, *Cornus*, *Corylus*, *Fagus*, *Fraxinus excelsior* type, *Hedera helix, Hippophae*, *Ilex*, *Juniperus*, *Lonicera*, *Olea*, *Ostrya*, *Phillyrea*, *Picea*, *Pinus*, *Pistacia*, *Platanus*, *Populus*, *Quercus* deciduous type, *Quercus* evergreen type, *Salix*, *Sorbus*, *Taxus*, *Tilia*, *Ulmus*, *Vitis*. In the main text, we selected the pollen records of some key tree species, *Pinus* (pine), *Quercus* (oak, deciduous + evergreen) and *Abies* (fir) and the total AP profile. Supplementary Figure S1 contains an AP record excluding *Pinus*, *Betula* and *Juniperus* to allow direct comparison with the AP record from the basin centre[14].

B biomarker proxy development and interpretation

Soil OM supply deduced from mid-chain suberin monomers and terrestrial fatty acid/alcohol ratios

The method of transesterification applied in this study (see above) methylates free fatty acids, but also breaks the ester bonds of extracted lipid polyesters such as cutin and suberin, releasing alkyl monomers. Both cutin and suberin form biological barriers in plants, with cutin

building a protective layer on the outside of epidermal cells of leaves while suberin is a major constituent of root tissue. An important compositional difference between cutin and suberin is the presence of mid-chain alkyl compounds in suberin with carbon chain lengths ≥ 20 while cutin is composed mainly of C_{16} and C_{18} alkyl compounds. In particular, *n*-alkanoic acids, *n*-alcohols and ω-hydroxy acids with chains formed of 22 or 24 carbon atoms are major suberin monomers[15]. Thus, variable amounts of mid-chain C_{22} and C_{24} compounds relative to long-chain (≥ C_{26}) plant wax-derived compounds are likely to indicate changes in the supply of soil OM, which can be expressed as the average chain length (ACL), e.g., for C_{22} to C_{26} *n*-fatty acids (FA) and *n*-alcohols (OH) using the general equation below (eq. 2).

$$\text{ACL FA}_{22\text{-}26} = (22 \times [n\text{-}C_{22}] + 24 \times [n\text{-}C_{24}] + 26 \times [n\text{-}C_{26}]) / ([n\text{-}C_{22}] + [n\text{-}C_{24}] + [n\text{-}C_{26}]) \quad (2)$$

A recent survey of the lipid composition of the major modern terrestrial and aquatic OM pools in the Ohrid Basin confirmed a decrease in ACL $FA_{22\text{-}26}$ from leaf litter to soils[16]. The survey also revealed that some of the topsoils and, in particular, the common Terra Rossa, are depleted in *n*-alkanoic acids relative to *n*-alcohols compared to leaf litter. Accordingly, the ratio of plant wax-derived long-chain (≥ C_{25}) *n*-alkanoic acids over *n*-alcohols provides a proxy for degraded soil OM supply relative to less degraded topsoil and plant litter.

$$\text{FA/OH}_{25\text{-}30} = \Sigma\, [n\text{-}C_{25\text{-}30}\ \text{FA}] / (\Sigma\, [n\text{-}C_{25\text{-}30}\ \text{FA}] + \Sigma\, [n\text{-}C_{25\text{-}30}\ \text{OH}]) \quad (3)$$

The proxy was initially named FA/OH_{terr}[15] but is renamed $FA/OH_{25\text{-}30}$ for consistency.

C Supporting proxy data (stable isotopes, Fig. S1)

Terrestrial habitat development

We determined the carbon isotope composition of the dominant *n*-alcohols, *n*-C_{22}, n-C_{26} and *n*-C_{28} OH, in particular, in samples between 140 and 109.9 ka in lower resolution. In order to determine if carbon isotopes differ between compounds formed using atmospheric CO_2 (leaf waxes) and soil CO_2 (root tissue) we calculated the difference between the suberin-derived *n*-C_{22} alcohol and the long-chain *n*-C_{26} and *n*-C_{28} alcohols which are common in leaf waxes (equation 5).

$$\Delta^{13}C = (\delta^{13}C_{26}\ \text{OH} + \delta^{13}C_{28}\ \text{OH}) / 2 - \delta^{13}C_{22}\ \text{OH} \quad (5)$$

Notably, the *n*-C_{22} alcohol appears between 4 and 6 ‰ less enriched in ^{13}C than the cuticular *n*-C_{26} and *n*-C_{28} alcohols between 124 and 117 ka (Fig. S1). This interval coincides with minimum values for bulk organic carbon isotopes determined previously[16]. Considering that CO_2 from microbial respiration tends to be depleted in ^{13}C, we hypothesize that the maximum difference between cuticular and root tissue lipids results from enhanced production of isotopically light CO_2 in the soils by aerobic microbial respiration and uptake during root tissue biosynthesis. This implies that soil respiration peaked during this period, suggesting maximum productivity and, in association with this, a maximum size of the soil organic matter pool. This would confirm the assumption that the lower bulk organic carbon isotope values during the Eemian/MIS 5d result from soil development in the catchment[17].

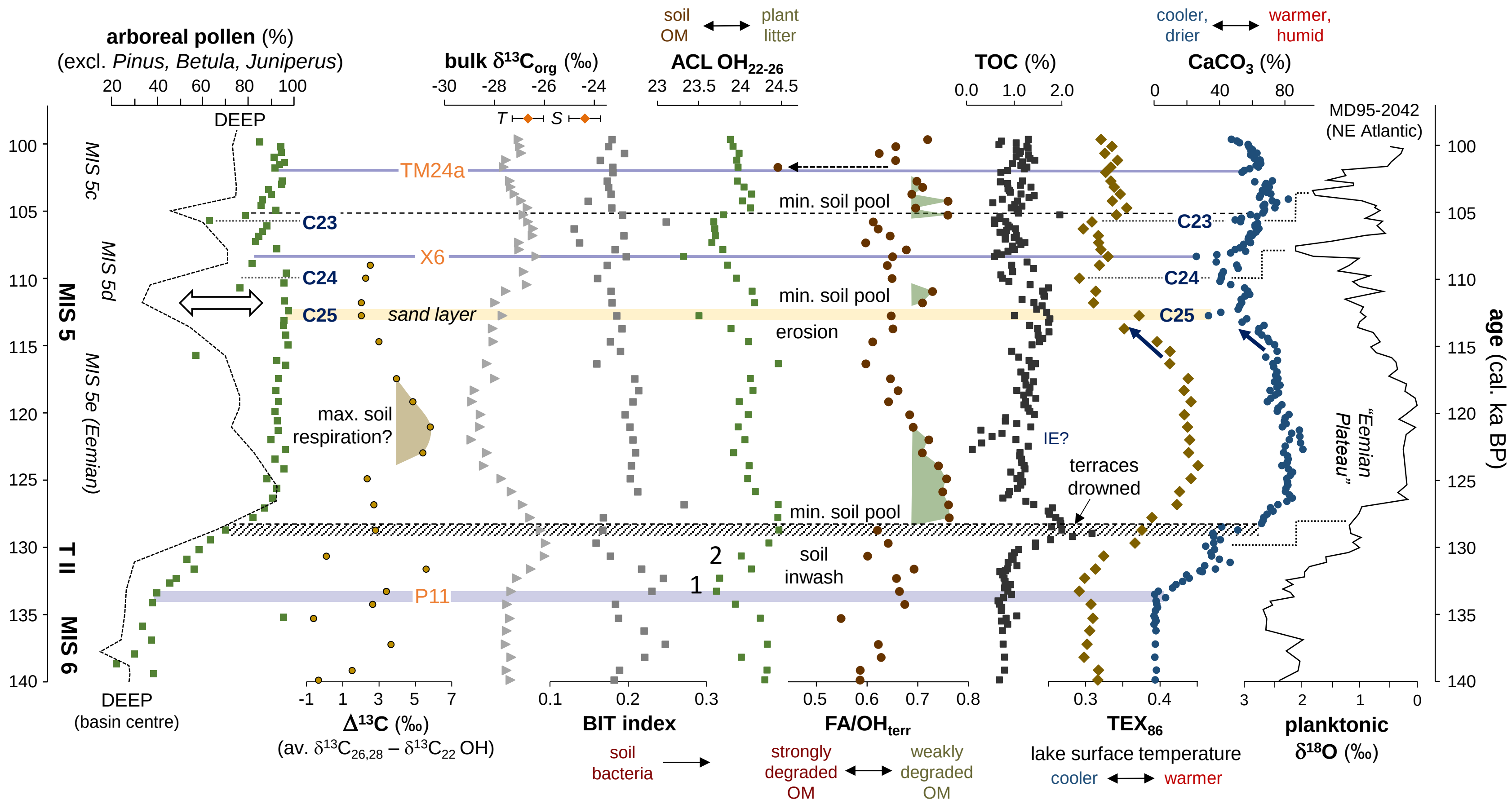

Figure S1: Palynological and organic geochemical data from site Co1202 (Ohrid Bay) from 140 to 99 cal. ka BP (for abbreviations and labels see Fig. 2 in main article). Additional data and proxy records from Co1202 include total organic carbon (TOC, in weight percent), ACL OH_{22-26}, the BIT index, bulk organic carbon isotopes[17] (bulk $\delta^{13}C_{org}$; *T* and *S* illustrate bulk $\delta^{13}C$ ranges of modern topsoils and subsoils, respectively) and the difference in compound-specific isotope values between leaf wax-derived *n*-alcohols (C_{26}, C_{28} OH) and supposedly mainly root-derived C_{22} OH ($\Delta^{13}C$ ‰). The temperature-sensitive proxies $CaCO_3$ and TEX_{86} are compared to planktonic $\delta^{18}O$ data from the NE Atlantic[18], illustrating the close relation between the climatic development in the North Atlantic realm and the Ohrid Basin. The record of arboreal pollen from Co1202 is compared to the corresponding record from the DEEP site[14] (basin centre; *Pinus*, *Betula* and *Juniperus* pollen excluded for comparison). Significant discrepancies between the basin centre and Co1202 during the late Eemian and MIS 5d, in particular, suggests that the catchment of Co1202 supported more forest-rich habitats during cold and dry intervals than the Ohrid Basin as a whole (block arrow highlights maximum discrepancy between the two sites). The discrepancies cannot be explained by selective transport as bisaccate pollen such as *Pinus* spp. are transported preferentially by wind[19]. Horizontal dashed lines represent the point in time at which the terrace surfaces as a source of soil OM appear to drown. Numbers 1 and 2 indicate the two consecutive phases of transgressive terrace erosion during the glacial-interglacial transition identified in steppic pollen and ACL FA_{22-23} records (Fig. 2, main article). The black dashed arrow highlights the impact that the deposition of the TM24a tephra apparently had on fatty acid (FA) preservation.

The BIT index indicates contribution of GDGTs from soil bacterial biomass to the sediment, thus, is assumed to reflect the variable supply of soil OM. With values < 0.3 throughout the record (0.19 on average) the BIT index suggests that supply of soil OM is minor. This is in stark contrast to alkyl and steroidal lipid data that, at times, indicates soil OM to be the major source for these compounds in Lake Ohrid sediments[16]. Variability of the BIT index is generally low and clearly differs from the profile of ACL OH_{22-26} that is assumed to reflect variable amounts of root-derived material. While the absolute values of the BIT index appear too low for a lacustrine sedimentary setting, some of its features do match those of the ACL OH_{22-26} profile, for example, during the first episode of transgressional erosion in the MIS 6-5 transition (phase 1) or at the onset of post-Eemian climate deterioration at 116 ka. Apart from two peaks during MIS 6 and the MIS 6-5 transition, a period of BIT values higher than 0.2 during MIS 5e, between 126 and 117 ka, also coincides with a period of relatively stable climate as indicated by other proxies (e.g., TEX_{86}), including ACL OH_{22-26}. The discrepancies between BIT and the alkyl lipid proxies (see also ACL FA_{22-26} in Fig. 2, main article) may partly be explained by variability in the amounts of bacterial biomass in different types of soil and/or climatically controlled changes in soil moisture, which is an important factor for bacterial activity and productivity in the soils. On the other hand, the proportion of root-derived mid-chain alkyl lipids may vary with soil type. In contrast to ACL OH_{22-26} and ACL FA_{22-26}, however, the BIT index has not been calibrated locally through the analysis of GDGTs of modern soils, while the low values remain unexplained. Furthermore, the alkyl lipid-based proxies show greater variability in association with known climatic changes. Finally, the isotopic composition of bulk organic carbon in the lake sediments[17] is consistent with those of modern soils from the Lake Ohrid catchment (Fig. S1), but not with the composition of common aquatic macrophytes that are significantly isotopically enriched relative to the topsoils and subsoils (Appendix 1B). Due to the uncertainties associated to the BIT index, we prioritise the interpretation of the alkyl lipid proxies.

References

1. Vogel, H. *et al*. A paleoclimate record with tephrochronological age control for the last glacial-interglacial cycle from Lake Ohrid, Albania and Macedonia. *J. Paleolimnol.* **44,** 295–310 (2010).
2. Leicher, N. *et al.* First tephrostratigraphic results of the DEEP site record from Lake Ohrid (Macedonia and Albania). **13,** 2151–2178 (2016).
3. Schouten, S. *et al*. Analytical methodology for TEX_{86} paleothermometry by high-performance liquid chromatography / atmospheric pressure chemical ionization-mass spectrometry. *Anal. Chem.* **79,** 2940–2944 (2007).
4. Schouten, S. *et al*. Distributional variations in marine crenarchaeotal membrane lipids: a new tool for reconstructing ancient sea water temperatures? *Earth Planet. Sci. Lett.* **204,** 265–274 (2002).
5. Hopmans, E. C. *et al.* A novel proxy for terrestrial organic matter in sediments based on branched and isoprenoid tetraether lipids. *Earth Planet. Sci. Lett.* **224,** 107–116 (2004).
6. Schouten, S. *et al*. The organic geochemistry of glycerol dialkyl glycerol tetraether lipids: A review. *Org. Geochem.* **54,** 19–61 (2013).
7. Weijers, J. W. H. *et al*. Occurrence and distribution of tetraether membrane lipids in soils: Implications for the use of the TEX_{86} proxy and the BIT index. *Org. Geochem.* **37,** 1680–1693 (2006).

8. Kiriakoulakis, K. Organic geochemistry of early diagenetic concretions. (PhD thesis University of Liverpool, 1997).

9. Stockmarr, J. Tablets with spores used in absolute pollen analysis. *Pollen et Spores* **13,** 614–621 (1971).

10. Bennett, K. D. & Willis, K. J. in *Tracking environmental change using lake sediments v. 3* (eds. Smol, J. P., Birks, H. J. B. & Last, W. M.) 5–32 (Kluwer Academic Publishers, 2001).

11. Moore, P. D. *et al*. *Pollen Analysis*. (Blackwell Scientific Publications, 1991).

12. Punt, W. *et al*. *The Northwest European Pollen Flora v. 1-9*. (Elsevier).

13. Reille, M. *Pollen et spores d'Europe et d'Afrique du nord. Supplement 1 and 2.* (Laboratoire de Botanique historique et Palynologie).

14. Sadori, L. *et al.* Pollen-based paleoenvironmental and paleoclimatic change at Lake Ohrid (south-eastern Europe) during the past 500 ka. *Biogeosciences* **13,** 1423–1437 (2016).

15. Molina, I. *et al*. The lipid polyester composition of Arabidopsis thaliana and Brassica napus seeds. *Phytochemistry* **67,** 2597–2610 (2006).

16. Holtvoeth, J. *et al.* Improved end-member characterisation of modern organic matter pools in the Ohrid Basin (Albania, Macedonia) and evaluation of new palaeoenvironmental proxies. *Biogeosciences* **13,** 795–816 (2016).

17. Leng, M. J. *et al.* Late Quaternary palaeoenvironmental reconstruction from Lakes Ohrid and Prespa (Macedonia / Albania border) using stable isotopes. 3109–3122 (2010). doi:10.5194/bg-7-3109-2010

18. Shackleton, N. J. *et al*. The classic marine isotope substage 5e. *Quat. Res.* **58,** 14–16 (2002).

19. Mudie, P. J. Pollen distribution in recent marine sediments, eastern Canada. *Can. J. Earth Sci.* **19**, 729–747 (1982).

Appendix 1: Biomarker and Stable Carbon Isotope Data

A) Co1202 biomarker proxies, $\Delta^{13}C$ of mid- and long-chain alcohols

**age** (cal. kyrs. BP)	**ACL** FA_{22-26}	**ACL** OH_{22-26}	FA/OH_{25-30}	TEX_{86}	**BIT**	$\Delta^{13}C$ (‰) (av. $\delta^{13}C_{26,28}$ - $\delta^{13}C_{22}$ OH)
99.539	24.7	23.9	0.72	0.32	0.18	-
100.057	24.3	23.9	0.66	0.34	0.17	-
100.575	24.3	24.0	0.62	0.33	0.20	-
101.093	24.2	24.0	0.66	0.34	0.16	-
101.611	24.2	24.0	0.42	0.33	0.18	-
102.000	-	-	-	0.33	0.18	-
102.648	24.7	24.0	0.70	0.33	0.17	-
103.101	24.6	24.0	0.71	0.34	0.17	-
103.619	24.6	24.1	0.69	0.35	0.18	-
104.137	24.6	24.0	0.76	0.34	0.15	-
104.655	24.7	24.1	0.70	0.36	0.18	-
105.173	-	23.8	0.76	0.34	0.19	-
105.691	24.3	23.7	0.61	0.31	0.25	-
106.209	24.4	23.7	0.62	0.30	0.13	-
106.727	24.3	23.7	0.65	0.32	0.19	-
107.245	24.3	23.7	0.60	0.32	0.14	-
107.763	24.3	23.8	0.68	0.32	0.18	-
108.281	24.0	23.3	0.65	0.33	0.20	-
108.934	24.3	23.8	0.64	0.32	0.17	2.5
109.903	24.4	24.0	0.65	0.29	0.16	2.3
110.871	24.6	24.1	0.73	0.31	0.18	-
111.730	24.6	24.2	0.71	0.31	0.18	2.0
112.698	24.1	23.5	0.65	0.37	0.19	2.0
113.666	24.1	23.9	0.65	0.35	0.19	-
114.634	24.4	24.1	0.61	0.40	0.18	3.0
115.360	-	-	-	0.41	0.19	-
116.280	24.6	24.5	0.60	0.41	0.16	-
117.393	24.5	24.1	0.65	0.44	0.21	4.0
118.277	24.4	24.2	0.66	0.43	0.21	-
119.112	24.3	24.0	0.64	0.44	0.21	4.9
120.080	24.5	24.1	0.68	0.43	0.20	-
120.999	24.3	24.0	0.69	0.44	0.20	5.8
121.955	24.3	24.1	0.72	0.44	0.21	-
122.924	24.3	23.9	0.71	0.44	0.21	5.4
123.892	24.6	24.1	0.74	0.45	0.20	-
124.860	24.6	24.1	0.76	0.44	0.20	2.3
125.828	24.7	24.2	0.75	0.43	0.21	-
126.796	24.8	24.5	0.76	0.42	0.27	2.7
127.764	24.7	24.5	0.76	0.39	0.17	-
128.696	24.4	24.5	0.62	0.38	0.17	2.8
129.664	24.0	24.3	0.64	0.37	0.16	-
130.632	24.1	24.0	0.60	0.32	0.18	0.1
131.600	24.9	24.1	0.69	0.31	0.22	5.6
132.290	24.2	23.8	0.66	0.30	0.24	-
133.258	24.3	23.7	0.66	0.29	0.23	3.4
134.226	24.5	23.9	0.67	0.31	0.18	2.6
135.279	24.5	24.2	0.55	0.31	0.19	-0.6
136.211	-	-	-	0.31	0.22	-
137.215	24.5	24.3	0.62	0.30	0.25	3.7
138.183	24.5	24.0	0.63	0.30	0.22	-
139.151	24.8	24.3	0.59	0.32	0.19	1.5
139.877	24.8	24.3	0.59	0.32	0.18	-0.3

B) soil and macrophyte bulk $\delta^{13}C_{org}$

	**bulk $\delta^{13}C_{org}$** (‰)	standard deviation	***n***
topsoils	-26.7	± 0.3	6
subsoils	-24.4	± 0.6	3
macrophytes	-18.0	± 3.7	6

Appendix 2: Pollen Data (data of individual *genera* in **% of total pollen count**)

		**arboreal pollen**									
**age** (cal. kyrs. BP)	***n*** (total pollen)	*Abies*	*Acer*	*Alnus*	*Betula*	*Buxus*	*Carpinus*	*Cornus*	*Corylus*	*Fagus*	*Fraxinus excelsior* type
99.734	616	14.6	0.3	-	-	-	0.3	-	0.2	0.6	-
100.122	531	38.4	-	0.2	-	0.2	1.7	-	-	2.3	-
100.511	513	43.2	-	-	-	0.4	0.8	-	0.2	3.1	-
100.899	508	29.5	0.2	0.2	0.2	0.2	1.7	-	0.2	3.7	-
101.288	606	40.4	-	-	-	0.2	1.6	-	0.2	5.1	0.2
101.417	541	33.4	0.2	0.2	-	-	1.1	-	0.2	3.7	-
101.676	511	27.6	0.4	0.2	-	-	2.3	-	-	0.8	-
102.670	606	31.0	0.3	-	-	-	1.5	-	0.3	2.6	-
102.907	519	33.0	-	-	-	-	2.9	-	-	2.7	-
103.295	618	23.3	0.2	-	-	-	1.9	-	-	1.9	-
103.684	534	27.4	0.4	0.2	-	-	2.0	-	-	1.5	-
104.072	516	17.6	0.8	0.2	-	-	1.9	-	0.2	1.7	-
104.461	544	13.7	-	0.2	0.4	-	1.6	-	0.2	0.4	-
104.849	613	30.2	0.2	-	-	-	1.4	-	-	0.3	-
105.238	522	7.4	0.6	0.2	-	-	0.6	-	0.2	1.3	-
105.626	505	10.9	0.2	-	-	-	-	-	0.4	0.4	-
106.015	522	20.4	0.2	-	0.2	-	0.2	-	0.6	0.6	-
106.403	640	22.8	0.3	-	-	-	0.5	-	0.2	0.6	-
106.792	516	21.1	1.0	-	-	-	0.4	-	0.2	0.6	-
107.180	510	24.1	0.2	0.4	-	-	0.4	-	-	0.6	-
107.698	515	26.7	-	-	0.2	-	0.2	-	-	0.4	-
108.813	448	19.9	-	-	0.2	0.2	0.0	-	-	-	-
109.540	509	44.7	-	0.2	0.2	0.2	2.2	-	0.2	1.0	-
110.266	600	38.1	-	-	-	-	1.0	-	-	0.3	-
110.629	513	23.7	-	0.2	0.2	-	0.6	-	-	-	-
111.609	552	46.7	-	-	0.2	0.2	1.6	-	-	0.2	-
112.335	519	39.3	0.2	0.2	-	-	2.1	-	-	-	-
113.061	634	39.6	0.2	0.3	-	0.2	5.4	-	-	0.2	-
113.424	513	47.5	0.2	-	-	0.2	4.5	-	0.2	-	-
114.150	512	34.2	-	0.2	-	0.4	9.1	-	0.4	-	-
114.876	598	50.9	-	0.2	-	0.2	3.2	-	0.2	0.3	-
115.675	382	1.7	-	-	-	-	0.3	-	-	-	-
116.038	520	21.1	-	0.4	0.2	-	1.9	-	-	-	-
116.401	605	24.7	-	0.2	-	-	2.8	-	-	-	-
117.393	604	18.3	-	-	-	-	2.5	-	-	-	-
118.277	655	19.4	0.1	0.1	-	0.1	2.7	-	-	-	-
119.112	639	24.5	0.5	-	0.2	-	2.0	-	0.2	0.2	-
119.838	503	26.0	-	-	-	0.6	2.9	-	-	-	-
120.564	526	24.1	-	-	-	0.2	2.1	-	-	0.2	-
121.241	507	23.9	0.2	-	-	-	1.5	-	-	-	0.2
121.955	546	12.4	0.2	-	-	-	0.4	-	-	-	-
122.681	515	17.8	0.2	0.2	-	-	0.4	-	0.2	-	0.2
123.408	526	18.8	0.2	0.4	-	-	0.2	-	0.2	0.2	-
124.134	546	16.7	0.5	0.4	-	-	0.4	0.2	0.2	-	0.2
124.860	524	12.3	0.7	-	-	-	0.2	-	0.2	-	-
125.586	545	15.7	-	-	-	-	0.4	-	-	0.2	0.4
126.312	541	12.8	0.4	-	-	-	-	-	-	-	-
127.038	576	14.1	0.2	-	-	-	-	-	0.2	0.3	-
127.764	510	14.8	0.2	-	-	-	0.2	-	-	0.2	-
128.696	462	12.4	-	-	-	-	0.2	-	-	0.2	-
129.422	495	4.0	0.2	0.2	-	-	0.2	-	-	0.2	-
130.148	451	0.9	-	-	0.4	-	-	-	-	0.2	-
130.874	414	0.9	0.2	-	-	-	-	-	-	0.5	-
131.600	427	1.6	-	-	-	-	-	-	-	-	-
132.290	413	1.3	-	-	-	-	0.2	-	-	0.7	-
132.653	409	0.5	0.2	-	1.0	-	-	-	-	0.2	-
133.379	425	0.8	-	-	0.5	-	-	-	0.2	-	0.2
134.105	413	0.5	-	-	0.2	-	-	-	0.2	-	-
135.194	604	18.2	-	-	-	0.3	3.9	-	-	-	-
135.848	419	0.7	-	-	-	-	-	-	-	-	-
136.900	436	0.6	-	-	0.2	-	-	-	-	-	-
137.941	423	1.4	-	-	0.5	-	-	-	0.2	-	-
138.667	503	0.3	-	-	0.2	-	0.2	-	-	-	-
139.393	530	0.7	-	-	0.2	-	-	-	-	-	-

Appendix 2, *continued*

age (cal. kyrs. BP)	arboreal pollen										
	Hedera helix	*Hippophae*	*Ilex*	*Juniperus*	*Lonicera*	*Olea*	*Ostrya*	*Phillyrea*	*Picea*	*Pinus*	*Pistacia*
99.734	-	-	-	-	-	-	-	-	-	67.4	-
100.122	-	-	-	-	-	-	-	-	0.8	46.3	-
100.511	-	-	-	-	-	-	-	-	0.7	39.8	-
100.899	-	-	-	-	-	-	-	-	0.4	47.8	-
101.288	-	-	-	-	-	-	-	-	0.2	47.4	-
101.417	-	-	-	-	-	-	-	-	0.7	47.0	-
101.676	-	-	-	-	-	-	-	-	0.5	52.5	-
102.670	-	-	-	-	-	-	-	-	0.2	51.7	-
102.907	-	-	-	-	-	-	-	-	0.2	45.0	-
103.295	-	-	-	-	-	-	-	-	-	54.7	-
103.684	-	-	-	-	-	-	-	-	0.4	47.2	-
104.072	-	-	-	-	-	-	-	-	0.2	46.6	0.4
104.461	-	-	-	-	-	-	0.2	-	-	48.9	-
104.849	-	-	-	-	-	-	-	-	0.1	50.6	-
105.238	-	-	-	0.2	-	-	-	-	-	69.3	-
105.626	-	-	-	0.2	-	-	-	-	0.1	61.1	-
106.015	-	-	-	-	-	-	-	-	0.4	50.4	-
106.403	-	-	-	-	-	-	-	-	0.5	51.9	-
106.792	-	-	-	-	-	-	-	-	0.7	55.2	-
107.180	-	-	-	0.2	-	-	-	-	1.2	46.2	-
107.698	-	-	-	-	-	-	-	-	0.6	58.7	-
108.813	-	-	-	1.1	-	-	-	-	0.7	63.8	-
109.540	-	-	-	-	-	-	-	-	2.4	38.0	-
110.266	0.2	-	-	0.2	-	-	-	-	2.6	48.7	-
110.629	-	-	-	0.2	-	-	-	-	2.7	54.9	-
111.609	-	-	-	-	-	-	-	-	5.0	36.9	-
112.335	-	-	-	-	0.2	-	-	-	5.2	46.1	-
113.061	-	-	-	-	-	-	-	-	0.9	40.9	-
113.424	-	-	-	0.2	-	-	-	-	0.2	37.6	-
114.150	-	-	0.2	-	-	-	-	-	0.9	42.6	-
114.876	0.2	-	-	-	-	-	-	-	1.4	37.6	-
115.675	-	1.1	-	1.1	-	-	0.3	-	0.3	86.6	-
116.038	0.2	-	-	-	-	0.2	-	-	0.4	65.2	-
116.401	0.2	-	-	-	-	-	-	-	-	62.0	-
117.393	-	-	-	-	-	-	-	-	0.1	71.3	-
118.277	-	-	-	0.1	-	-	-	0.1	0.2	67.2	-
119.112	-	-	-	-	-	-	-	-	-	63.1	-
119.838	-	-	-	-	-	-	-	-	-	55.0	-
120.564	-	-	-	0.4	-	-	-	-	-	56.0	-
121.241	-	-	-	-	-	-	-	-	-	52.3	-
121.955	0.2	-	-	-	-	-	-	0.2	-	66.7	-
122.681	0.2	-	-	-	-	-	-	0.2	-	46.8	-
123.408	0.2	-	-	-	-	-	-	-	-	47.9	0.2
124.134	-	-	-	0.2	-	-	-	-	-	51.5	0.2
124.860	-	-	-	0.2	-	-	-	-	-	59.2	0.2
125.586	0.2	-	-	0.2	-	-	-	-	-	42.4	0.2
126.312	-	-	-	0.2	-	0.2	-	-	-	49.5	0.4
127.038	0.2	-	-	-	-	-	-	-	-	59.0	-
127.764	-	-	-	0.2	-	-	-	-	-	54.5	0.2
128.696	0.2	-	-	0.2	-	-	-	-	0.1	55.5	0.4
129.422	-	-	-	0.2	-	-	-	-	-	70.6	-
130.148	-	0.9	-	1.5	-	-	-	-	-	66.1	-
130.874	-	-	-	1.4	-	-	-	-	-	64.5	-
131.600	-	0.2	-	0.7	-	-	-	-	0.1	65.9	-
132.290	-	0.2	-	-	-	-	-	-	0.2	56.0	-
132.653	-	-	-	2.2	-	-	-	-	-	50.4	-
133.379	-	0.7	-	1.5	-	-	-	-	-	64.3	-
134.105	-	0.5	-	0.5	-	-	-	-	-	68.6	-
135.194	0.2	-	-	-	-	-	-	-	-	67.9	-
135.848	-	0.7	-	-	-	-	-	-	0.2	86.9	-
136.900	-	0.7	-	0.2	-	-	-	-	-	89.9	-
137.941	-	0.2	-	0.7	-	-	-	-	0.2	82.9	-
138.667	-	0.6	-	-	-	-	-	-	0.4	81.9	-
139.393	-	0.5	-	1.1	-	-	-	-	0.3	86.7	-

Appendix 2, *continued*

	**arboreal pollen**										**steppic p.**
**age** (cal. kyrs. BP)	*Platanus*	*Populus*	*Salix*	*Sorbus*	*Taxus*	*Tilia*	*Ulmus*	*Quercus* deciduous	*Quercus* evergreen	*Vitis*	*Artemisia*
99.734	-	-	-	-	-	-	0.2	11.4	0.3	-	-
100.122	-	-	-	-	-	-	0.2	6.6	0.2	-	0.2
100.511	-	-	-	-	-	-	0.2	8.1	0.2	-	0.6
100.899	0.2	-	-	-	-	-	0.8	10.1	0.2	-	0.6
101.288	-	-	-	-	-	0.3	0.2	2.3	-	-	-
101.417	-	-	-	-	-	0.2	0.7	8.6	0.7	-	-
101.676	-	-	-	-	-	0.2	0.2	11.3	0.4	-	-
102.670	-	-	-	-	-	0.2	0.3	9.0	0.5	-	0.2
102.907	-	-	-	-	-	-	0.2	12.4	0.6	-	0.2
103.295	-	-	-	-	-	0.2	0.2	12.9	-	-	0.3
103.684	-	-	-	-	-	0.6	0.2	15.2	-	-	1.1
104.072	-	-	0.2	-	-	0.2	-	22.0	0.8	-	1.4
104.461	-	0.2	-	-	-	0.7	0.4	25.3	0.4	-	0.9
104.849	-	-	-	-	-	0.3	-	13.0	-	-	-
105.238	-	-	-	-	-	0.2	0.2	13.5	-	-	0.6
105.626	-	-	-	-	-	-	0.2	12.1	0.2	-	3.0
106.015	-	-	-	-	-	0.4	0.2	20.3	0.4	-	0.8
106.403	-	-	-	-	-	0.2	0.2	16.6	-	-	-
106.792	-	-	-	-	-	0.2	0.2	13.4	0.2	-	0.4
107.180	-	-	-	-	-	0.8	0.2	16.3	0.4	-	1.5
107.698	-	-	-	-	-	0.2	0.2	9.3	0.6	-	-
108.813	-	-	-	-	-	-	-	7.4	0.2	-	0.2
109.540	-	-	-	-	-	0.4	-	8.4	0.4	-	0.4
110.266	-	-	-	-	-	-	0.3	6.5	-	-	-
110.629	-	-	-	-	-	-	-	6.9	-	-	0.8
111.609	-	-	-	0.2	0.2	0.2	0.2	5.8	-	-	0.2
112.335	-	-	-	-	-	0.4	-	4.6	0.6	-	0.2
113.061	-	-	-	-	0.2	-	0.6	8.9	0.5	-	-
113.424	-	-	-	-	-	-	0.4	5.6	1.0	-	-
114.150	-	-	-	-	-	-	-	9.1	1.2	-	-
114.876	-	-	-	-	-	0.2	0.2	3.5	0.7	-	0.2
115.675	-	-	-	-	-	-	-	3.4	-	-	0.5
116.038	-	-	-	-	-	-	-	7.2	0.8	-	-
116.401	-	-	-	-	-	0.2	0.2	7.7	0.5	-	0.2
117.393	-	-	-	-	-	0.3	0.2	4.9	0.5	-	-
118.277	-	-	-	-	-	0.9	0.0	5.8	0.3	-	-
119.112	-	-	-	-	-	0.5	-	6.6	0.2	-	-
119.838	-	-	-	-	-	0.4	0.2	10.9	0.6	-	-
120.564	-	-	-	-	-	2.1	0.2	10.3	1.1	0.2	0.2
121.241	-	-	-	-	-	0.8	-	16.8	1.0	-	-
121.955	-	-	-	-	0.2	2.1	0.4	13.6	-	-	-
122.681	-	-	-	-	-	0.8	0.4	29.2	1.0	-	-
123.408	-	-	-	-	-	2.2	0.6	23.8	0.7	-	-
124.134	-	-	-	-	-	4.9	0.2	21.9	0.5	-	-
124.860	-	-	-	-	-	3.2	-	17.8	0.2	-	-
125.586	-	-	-	-	-	1.3	0.4	34.1	0.5	-	0.2
126.312	-	-	0.2	-	-	2.2	-	29.0	0.5	-	-
127.038	-	-	-	-	-	2.9	0.8	16.7	0.2	-	-
127.764	-	-	-	-	-	2.6	-	18.6	0.4	-	0.2
128.696	-	-	-	-	-	1.0	0.6	15.4	0.8	-	-
129.422	-	-	-	-	-	-	0.8	12.8	0.2	-	0.4
130.148	-	-	-	-	-	-	0.7	16.1	-	-	3.5
130.874	-	-	0.2	-	-	-	-	14.8	0.2	-	3.9
131.600	-	-	-	-	-	-	-	16.6	-	-	0.7
132.290	-	-	-	-	-	-	-	18.4	-	-	0.7
132.653	-	-	-	-	-	-	0.2	19.5	0.2	-	6.6
133.379	-	-	-	-	-	-	0.2	10.7	-	-	3.9
134.105	-	-	-	-	-	-	-	10.4	-	-	4.6
135.194	-	-	-	-	-	-	0.2	7.5	0.5	-	-
135.848	-	-	-	-	-	-	-	2.7	-	-	0.5
136.900	-	-	-	-	-	-	-	2.1	-	-	0.9
137.941	-	-	-	-	-	-	-	2.6	-	-	1.4
138.667	-	-	-	0.2	-	-	-	2.2	-	-	1.0
139.393	-	-	-	-	-	-	-	2.9	-	-	0.2

Appendix 2, *continued*

age (cal. kyrs. BP)	steppic pollen *Chenopodiaceae*	*Ephedra distachya* type	*Ephedra fragilis type*	grasses ***Poaceae***	**steppic**	**AP**	**PAR** (cm^{-2} yr^{-1}, in 1,000s)
99.734	-	-	-	1.3	-	95.2	1.6
100.122	0.2	-	-	0.4	0.4	96.8	1.9
100.511	0.6	-	-	1.0	1.2	96.7	1.4
100.899	0.4	-	-	1.2	1.0	95.3	1.1
101.288	-	-	-	0.8	-	98.0	2.1
101.417	-	-	-	0.9	-	96.7	0.8
101.676	-	-	-	1.8	-	96.3	1.2
102.670	-	-	-	0.5	0.2	97.7	1.4
102.907	0.2	-	-	0.8	0.4	96.9	1.2
103.295	0.2	-	-	1.9	0.5	95.2	1.7
103.684	0.2	-	-	1.7	1.3	95.0	1.3
104.072	0.6	-	-	2.9	1.9	92.7	0.9
104.461	0.2	0.4	-	2.7	1.5	92.5	0.8
104.849	-	-	0.2	0.5	0.2	96.1	1.3
105.238	0.2	-	-	2.3	0.8	93.6	0.9
105.626	1.4	-	-	7.1	4.4	85.7	0.7
106.015	0.2	0.2	-	3.0	1.1	94.1	1.0
106.403	-	-	-	2.0	-	93.6	0.9
106.792	0.4	-	0.2	1.9	1.0	93.1	1.3
107.180	1.3	-	0.2	2.1	3.1	90.8	0.9
107.698	0.2	-	-	1.6	0.2	97.1	1.8
108.813	0.7	-	0.2	2.7	1.1	93.5	1.1
109.540	0.2	-	-	0.8	0.6	98.2	4.2
110.266	-	-	-	0.7	-	97.8	2.7
110.629	1.2	0.2	-	5.3	2.2	89.4	0.6
111.609	-	0.2	0.2	1.6	0.5	97.3	3.8
112.335	-	-	0.2	0.2	0.4	98.8	3.7
113.061	-	-	-	0.6	-	97.7	1.3
113.424	-	-	-	1.4	-	97.5	1.9
114.150	-	-	-	0.2	-	98.1	1.8
114.876	-	-	-	0.3	0.2	98.5	2.4
115.675	0.3	-	-	2.1	0.8	94.7	0.5
116.038	0.2	-	-	0.6	0.2	97.5	2.9
116.401	-	-	-	0.0	0.2	98.4	2.4
117.393	-	-	0.2	0.2	0.2	98.0	3.4
118.277	-	-	-	0.3	-	97.3	4.0
119.112	-	-	-	0.0	-	97.7	2.1
119.838	-	-	-	0.4	-	96.5	1.1
120.564	-	-	-	0.2	0.2	96.8	2.0
121.241	-	-	-	0.6	-	96.7	1.5
121.955	-	-	-	-	-	96.2	1.4
122.681	-	-	-	0.6	-	97.5	1.1
123.408	0.2	-	0.2	0.9	0.4	95.5	0.9
124.134	-	-	-	0.5	-	98.0	1.0
124.860	0.2	-	0.2	0.2	0.4	94.2	1.0
125.586	0.9	-	-	0.5	1.1	95.8	1.2
126.312	-	-	-	1.3	-	95.3	0.9
127.038	-	-	0.3	1.0	0.3	94.6	1.2
127.764	-	-	-	0.4	0.2	91.8	1.4
128.696	-	-	-	0.8	-	87.0	1.3
129.422	-	-	-	2.3	0.4	89.3	1.3
130.148	1.3	-	-	2.6	4.8	86.7	1.2
130.874	2.8	0.9	-	2.1	7.7	82.8	0.8
131.600	3.6	0.2	-	2.7	4.5	85.2	0.6
132.290	2.8	0.2	-	5.6	3.7	77.0	0.5
132.653	3.9	-	-	8.5	10.5	74.5	0.4
133.379	2.4	0.5	-	7.5	6.8	79.2	0.3
134.105	1.2	-	-	5.6	5.8	80.9	0.3
135.194	-	-	-	-	-	98.7	3.1
135.848	0.7	-	-	4.6	1.2	91.3	0.3
136.900	0.2	0.2	-	2.3	1.4	93.8	0.6
137.941	0.5	-	-	5.9	1.9	88.8	0.4
138.667	-	-	-	8.6	1.0	86.0	0.3
139.393	0.2	-	-	2.0	0.4	92.5	0.3
